# Recent progress in targeted therapy for non-small cell lung cancer

**DOI:** 10.3389/fphar.2023.1125547

**Published:** 2023-02-21

**Authors:** Yanxia Xiao, Pu Liu, Jie Wei, Xin Zhang, Jun Guo, Yajun Lin

**Affiliations:** ^1^ The Key Laboratory of Geriatrics, Beijing Institute of Geriatrics, Institute of Geriatric Medicine, Chinese Academy of Medical Sciences, Beijing Hospital, National Center of Gerontology of National Health Commission, Beijing, China; ^2^ Peking University Fifth School of Clinical Medicine, Beijing, China

**Keywords:** non-small cell lung cancer, NSCLC, targeted therapy, drug resistance, CLIP1-LTK

## Abstract

The high morbidity and mortality of non-small cell lung cancer (NSCLC) have always been major threats to people’s health. With the identification of carcinogenic drivers in non-small cell lung cancer and the clinical application of targeted drugs, the prognosis of non-small cell lung cancer patients has greatly improved. However, in a large number of non-small cell lung cancer cases, the carcinogenic driver is unknown. Identifying genetic alterations is critical for effective individualized therapy in NSCLC. Moreover, targeted drugs are difficult to apply in the clinic. Cancer drug resistance is an unavoidable obstacle limiting the efficacy and application of targeted drugs. This review describes the mechanisms of targeted-drug resistance and newly identified non-small cell lung cancer targets (e.g., KRAS G12C, NGRs, DDRs, CLIP1-LTK, PELP1, STK11/LKB1, NFE2L2/KEAP1, RICTOR, PTEN, RASGRF1, LINE-1, and SphK1). Research into these mechanisms and targets will drive individualized treatment of non-small cell lung cancer to generate better outcomes.

## Introduction

With the population aging, the main cause of death for human beings has changed from infectious diseases to chronic diseases, and cancer is a type of chronic disease that seriously endangers the health of the population (Bauer et al., 2014; Xia et al., 2022). Among malignant tumors, lung cancer morbidity ranks second, its mortality ranks first, and nearly 85% of lung cancer cases are non-small cell lung cancer (Bray et al., 2018; Melosky et al., 2021; Sung et al., 2021; Xia et al., 2022). Therefore, Effective non-small cell lung cancer treatment must be sought immediately. The current treatment methods for non-small cell lung cancer include mainly surgery, chemotherapy, radiotherapy, targeted therapy and immunotherapy. Unfortunately, the majority of patients are diagnosed with advanced lung cancer, and the 5-year relative survival rate of patients (57%) diagnosed with metastatic lung cancer is 6% (Siegel et al., 2021). Traditional surgery and chemoradiotherapy show limited efficacy in these patients. Because therapeutic medications are consistently directed to the identified cancer-causing locations, targeted therapy is a form of treatment that specifically picks cancer-causing sites at the cellular molecular level, killing tumor cells while sparing healthy cells. With the advent of targeted therapies, the prognosis of patients with NSCLC has profoundly improved, and a series of clinical trials have shown that the progression-free survival (PFS) of patients who receive drugs that target oncogenic sites has been greatly extended compared with that associated with chemotherapy drugs. The FDA has approved drugs that target epidermal growth factor receptor (EGFR), anaplastic lymphoma kinase (ALK), c-ros oncogene 1 (ROS1), rearranged during transfection (RET), the mesenchymal-epithelial transition (MET), neurotrophic tropomyosin tyrosine kinase (NTRK), and V-Raf murine sarcoma viral oncogene homolog (BRAF) for the clinical treatment in NSCLC patients (Melosky et al., 2021). Despite the results of targeted therapies, drug resistance is inevitable (Thai et al., 2021). Addressing drug resistance at important targets and finding new therapeutic targets are top priorities for the systematic treatment of NSCLC. In this review, we describe the advancements made in our understanding drug resistance mechanisms at these important targets, some of the newly discovered targets that play an important role in NSCLC, and the development of drugs that target these sites, which may be of great help in the treatment of NSCLC.

## Progress in targeted therapy resistance of NSCLC

**FIGURE 1:**
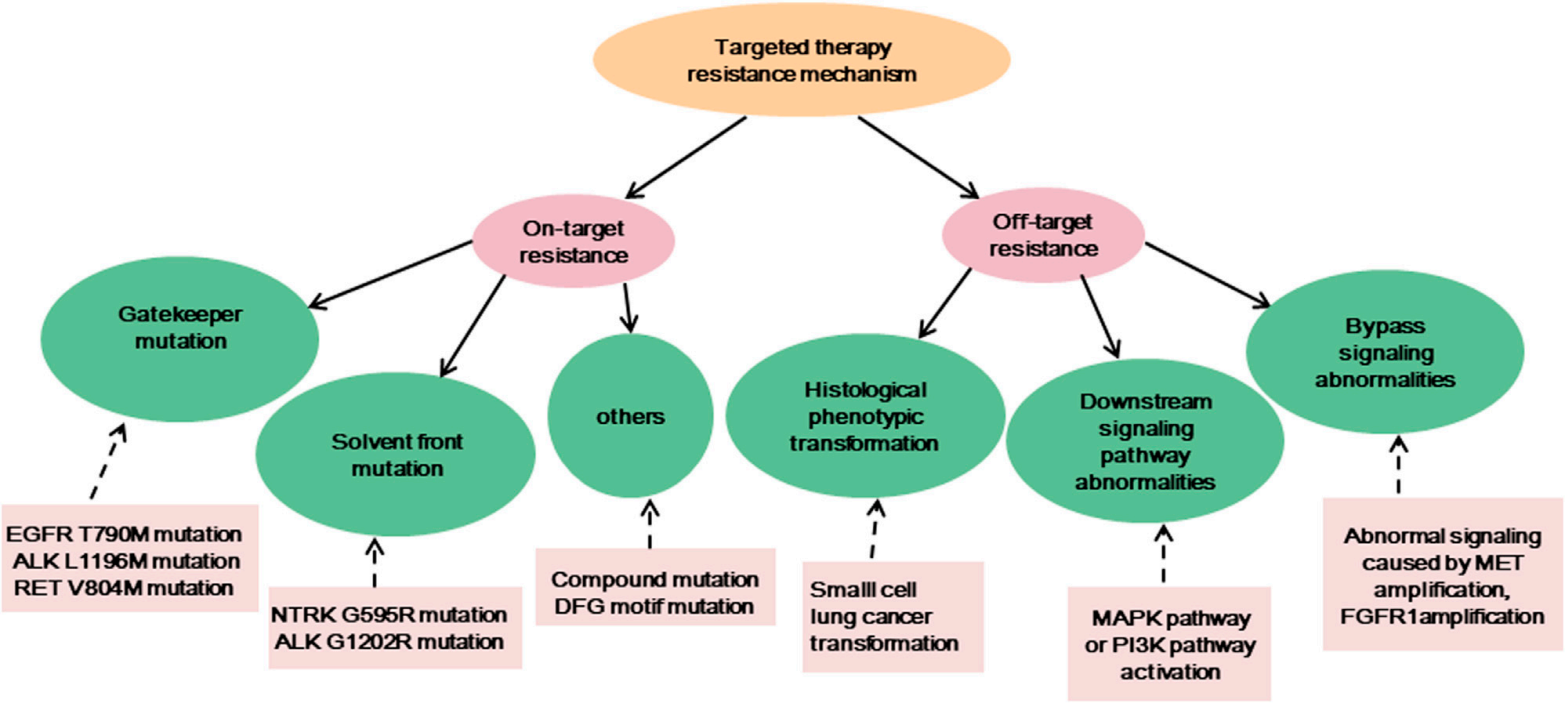
The resistance mechanisms of existing targeted therapies, and the examples of corresponding mechanisms.

Targeted therapy is currently one of the main means of treating advanced cancer. Although the treatment effect is profound, the tumor initiates many resistance mechanisms, and drug treatment eventually makes the tumor resistant to the drugs, reducing their efficacy. One of the main factors contributing to the death from cancer is drug resistance. The resistance of cancer cells to targeted drugs is an urgent problem in current cancer treatment (Table 1). In the current review, the mechanism of targeted therapy resistance is classified into two types: on-target resistance and off-target resistance (Figure 1) (Thai et al., 2021). On-target resistance is mainly due to changes in target structure that prevent targeted drugs from binding to sites. Off-target resistance includes downstream signaling pathway abnormalities (RAS-MAPK signaling pathway and PI3K signaling pathway activation), bypass signaling abnormalities (abnormal signaling caused by MET amplification), and histological phenotypic transformation.

**TABLE 1:** Resistance mechanisms of approved targeted therapy.

Gene name	Target drugs clinically used	Drug resistance mechanisms	Methods to overcome the resistance
EGFR	Gefitinib, erlotinib, afatinib, dacomitinib, icotinib, osimertinib	Devoleping new EGFR mutations like T790M and C797S mutation	Identify the causes of drug resistance with biopsy and Select appropriate EGFR-TKI or drug combination therapy.
		Downstream or bypass signaling pathway abnormalities	
		Histological phenotypic transformation	
ALK	Crizotinib, alectinib, ceritinib, ensartinib, brigatinib, lorlatinib	Devoleping new ALK mutations like G1202R mutation	ALK -TKI Sequential therapy
		Bypass signaling pathway abnormalities	
ROS1	Crizotinib, entrectinib	Devoleping new ROS1 mutations like G2032R mutation	Identify the causes of drug resistance with biopsy, then change the drug like Lorlatinib, Ropotrectinib or drug combination therapy
RET	Selpercatinib, pralsetinib	Devoleping new RET mutations	Chemotherapy, combination therapy of EGFR inhibitors and MET inhibitors
		MET/MYC amplification	
MET	Tepotinib, savolitinib, crizotinib, cabozantinib	Devoleping new MET mutations like D1288 and Y1230 mutations	Combination therapy with EGFR-TKI
		Gene amplification of EGFR, FGFR1, and KRAS.	
NTRK	Larotrectinib, entrectinib	Solvent front mutations such as G595R	Select the second-generation TRK inhibitors such as Selitrectinib and repotrectinib
		KRAS mutation, MET amplification, BRAF mutation, or IGF1R activation	
BRAF	Villafinil, dabrafinib, connephinil	Reactivation of the PI3K-AKT-mTOR and RAS-RAF-MEK pathways	Combination therapy of MEK inhibitors and BRAF inhibitors

EGFR is a transmembrane tyrosine kinase-receptor that mediates epidermal growth factor (EGF)-induced cell proliferation and signaling and belongs to the ErbB receptor family that comprises four similar related proteins (ErbB1–4) (Sankar et al., 2020; Li et al., 2022). EGFR mutations are the most common driver mutation in NSCLC, and approximately50% of Asian patients with adenocarcinoma carry EGFR mutations (Shi et al., 2014). The current gold standard of treatment for advanced NSCLC with EGFR mutations is EGFR-targeted medication therapy. Even while EGFR tyrosine kinase inhibitors (TKIs) initially work well for the majority of people with EGFR mutations, almost all patients eventually experience disease progression due to acquired resistance to the targeted drugs. After 12 months of using first- and second-generation inhibitors, 60% of patients developed the drug-resistant mutation T790M, which reduces drug efficacy by altering the structural domain of the kinase (Li et al., 2022). Third-generation EGFR-TKIs attenuate the resistance induced by the EGFR T790M mutation, but new mutations in EGFR are generated after treatment, such as C797S and G796X (Li et al., 2022). Currently, to combat mutations like C797S and T790M, fourth-generation EGFR-TKIs are being created. BLU-945 and OBXOZ-011 have shown good results against C797S and T790M mutants in preclinical studies (Eno et al., 2022). In addition, combination therapy can overcome resistance caused by C797S mutations. Brigatinib and cetuximab combination prolongs median PFS compared with chemotherapy alone in NSCLC patients with EGFR-activating mutation, T790M, and cis-C797S triple mutations (Wang et al., 2020). Combination therapy of first- and third-generation EGFR-TKI could overcome the resistance of EGFR 19Del/T790M/in trans-C797S (Zhou et al., 2019). Another cause of resistance to EGFR-TKIs is abnormal activation of bypass signaling, such as MET amplification; RET rearrangement; NTRK rearrangement; BRAF rearrangement/mutation; abnormalities in downstream signaling pathway targets resulting in the abnormal activation of EGFR downstream signaling pathways, such as KRAS; PIK3CA point mutations; or histological phenotypic transformation (Yang et al., 2022).

ALK is a transmembrane receptor tyrosine kinase and is a member of the insulin receptor superfamily and is crucial for the growth and operation of the nervous system, and in most normal cells, ALK is inactive (Morris et al., 1994). ALK fusion is clinically more common in young adenocarcinoma patients who do not smoke or smoke infrequently, and the most common type of fusion is EML4-ALK (Franco et al., 2013). ALK-TKIs are highly effective in patients with advanced ALK-positive NSCLC, and several ALK-TKIs have been approved for marketing and for first-line treatment of patients with ALK-fusion NSCLC. Mutations in the ALK kinase domain are the main cause of secondary resistance to ALK-targeted treatment, and G1202R is the most common drug-resistant mutation that occurs during disease progression. Other common mutations include L1196 M, G1269A C1156Y, I1174T/S, S1206Y, E1210K, F1174C/L, and V1180 L (Recondo et al., 2020; Lin et al., 2021). Furthermore, novel compound ALK mutations and NF2 loss-of-function mutations have been reported to be associated with drug resistance in a few cases (Recondo et al., 2020). TPX-0131 and NUV-655 are two-fourth generation ALK-TKIs that are currently in development. TPX-0131 and NUV-655 can suppress compound ALK mutations in addition to broad-spectrum single ALK mutations (Ou et al., 2021a). Off-target resistance is not a common cause of ALK-TKI resistance.

ROS1 is a member of the transmembrane tyrosine kinase receptor family. The fused ROS1 protein loses most of its extracellular structural domain and is continuously activated without ligand binding, resulting in the abnormal activation of downstream signaling pathways. The incidence of ROS1 mutations in NSCLC is 1%–2% and is more common in patients who do not smoke or infrequently smoke and in those with adenocarcinoma (Drilon et al., 2021). The ROS1 gene can undergo abnormal fusion with multiple genes, with the predominant fusion partner being CD74 (Drilon et al., 2021). In addition, the ROS1 fusion does not coexist with other driver genes. Crizotinib was the first FDA-approved targeted drug for ROS1-positive NSCLC (Pathak et al., 2021). In ROS1-targeted therapy in drug-resistant patients, G2032R is the most common resistance mutation, and other resistance mutations include G1957A, S1986F, and G2086F (Drilon et al., 2021). ROS1/TRK/ALK inhibitors (repotrectinib and taletrectinib) have shown early clinical efficacy against G2032R mutation-induced resistance (Drilon et al., 2021). Off-target resistance mechanisms of ROS1-TKIs are less common.

RET, is a transmembrane receptor belonging to the tyrosine protein kinase family. RET rearrangement accounts for approximately 1%–2% of NSCLC, mainly in patients with a no-smoking or light-smoking history and adenocarcinoma (Lin et al., 2020). RET rearrangements do not typically overlap with EGFR, ROS1, BRAF, MET exon 14 skipping, or ALK genetic variants. Tyrosine kinase inhibitors with anti-RET activity are effective in treating patients with lung cancer caused by RET rearrangement. The FDA approved the RET selective inhibitor, such as selpercatinib (LOXO-292) and pralsetinib (BLU-667), as the standards of care for RET-rearrangement-involved advanced NSCLC (Lin et al., 2020). One mechanism of RET inhibitor resistance is RET solvent front mutation, and the RET G810R/S/C/V mutant mediates acquired resistance to selpercatinib (Lin et al., 2020). However, the resistance mechanism of selective RET inhibitors still needs to be further explored. Resistance to RET inhibitors is often caused by non-RET-dependent resistance, such as acquired MET and KRAS amplification. Histologic type transformation is uncommon.

MET encodes hepatocyte growth factor receptor (HGFR), also referred to as c-MET, which is a transmembrane receptor with autonomous phosphorylation activity that belongs to the tyrosine kinase receptor superfamily and is mainly expressed in epithelial cells (Organ and Tsao, 2011). In NSCLC, the overall incidence of MET exon 14-skipping mutations is approximately 3%–5.6%, and these mutations do not coexist with other NSCLC driver variations, like ALK or EGFR (Frampton et al., 2015; Heist et al., 2016). Although MET inhibitors, as represented by tepotinib and savolitinib, show good antitumor effects, resistance to MET inhibitors is inevitable. MET-TKIs can be classified into 3 types (Type I, Type II and Type III) (Gherardi et al., 2012). Type I TKIs are competitive inhibitors of ATP and are further classified into Type Ia and Type Ib TKIs. The commonly used clinical drug crizotinib is a Type Ia MET-TKI, and tepotinib, savolitinib and AMG337 are Type Ib MET-TKIs. Type II MET-TKIs are generally multitarget TKIs, and cabozantinib is a Type II MET-TKI. Type III MET-TKIs act on allosteric sites that are completely different from ATP-binding sites, and none of these drugs has been entered into the clinical research stage (Reungwetwattana et al., 2017). D1288 and Y1230 mutations in MET genes may lead to Type I MET-TKI resistance, and L1195 and F1200 mutations may lead to Type II MET-TKI resistance (Fujino et al., 2019; Koga et al., 2022). In addition, gene amplification of EGFR, fibroblast growth factor receptor 1 (FGFR1), and KRAS is an off-target resistance mechanism (Han et al., 2019).

NTRK contains NTRK1, NTRK2, and NTRK3, which respectively encode TRKA, TRKB, and TRKC (Koga et al., 2022; Manea et al., 2022). First-generation TRK inhibitors larotrectinib and entrectinib are currently the drugs of choice for patients with NTRK-fusion tumors (Liu et al., 2022; Manea et al., 2022). Solvent front mutations, such as G595R, G623R, and G667C, are the causes of the most common type of resistance. This resistance mechanism can be reversed with second-generation TRK inhibitors, and the two main drugs currently under development are selitrectinib (LOXO-195) and repotrectinib (TPX-0005). NTRK inhibitor off-target resistance mainly includes bypass or downstream pathway signaling activation caused by KRAS mutation, MET amplification, BRAF mutation, or IGF1R activation.

BRAF belongs to the RAF family, and the BRAF gene is critical for encoding a RAF kinase protein involved in the RAS-RAF-MEK-ERK signaling pathway (Tabbo et al., 2022). Roughly 4% of NSCLC cases are BRAF-mutant lung cancer instances (Tabbo et al., 2022). Among NSCLC patients carrying BRAF mutations, approximately 50% consist of the BRAF V600E mutant. BRAF V600E inhibitors, represented by villafinil, dabrafinib, and connephinil show high inhibitory activity against BRAF mutants, especially the BRAF V600E mutant (Tabbo et al., 2022). Reactivation of the PI3K-AKT-mTOR and RAS-RAF-MEK pathways is the main resistance mechanism that is targeted BRAF V600E-mutant NSCLC therapy (Tabbo et al., 2022). Other mechanisms of resistance have yet to be studied.

## Emerging targets

**FIGURE 2:**
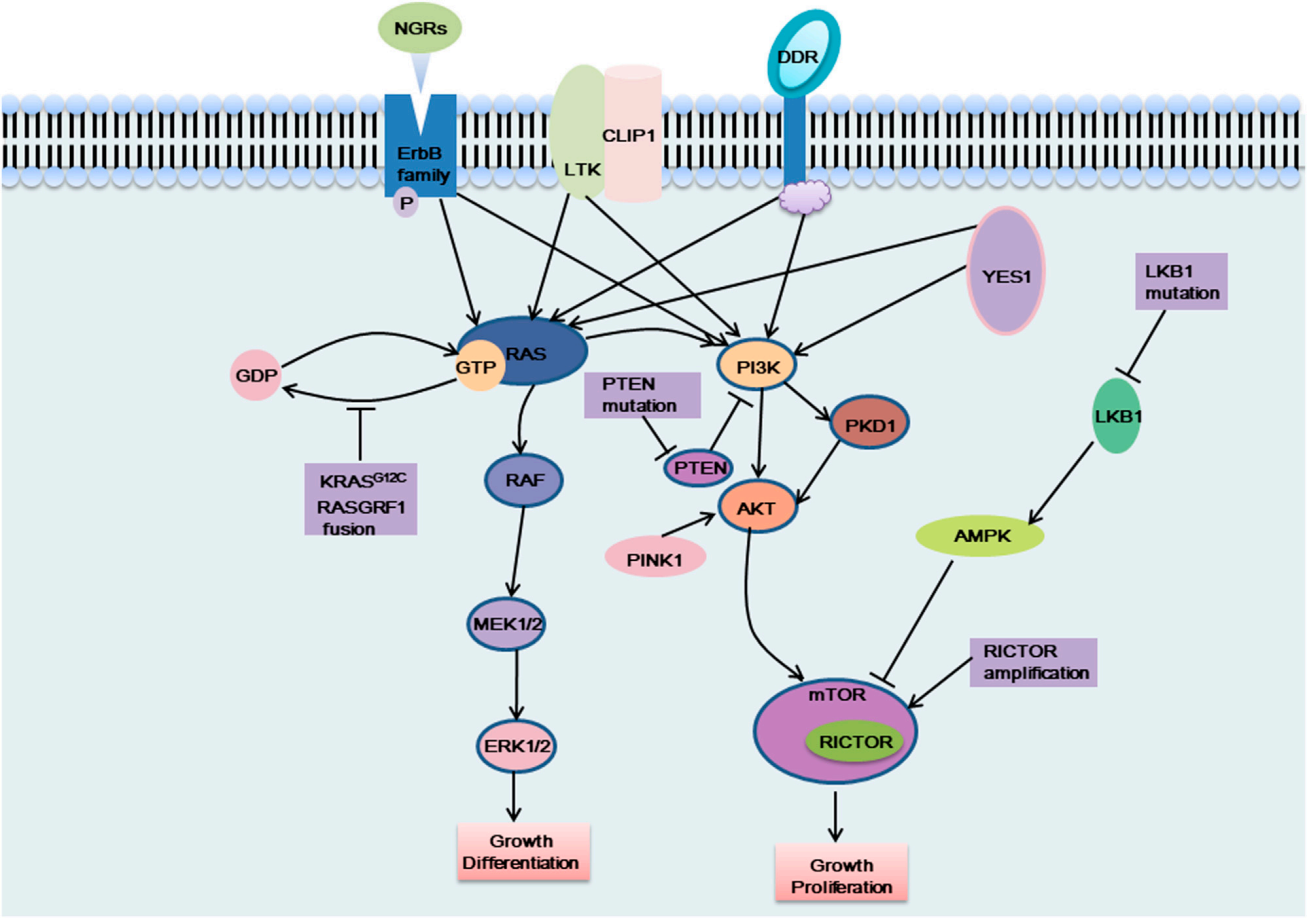
The carcinogenic pathways were affected by these emerging targets. NGRs, CLIP1-LTK fusion, DDR mutation and YES1 activate the MAPK pathway and the PI3K pathway, which can affect cell proliferation and differentiation, etc. KRAS G12C mutation and RASGRF1 fusion affect the dissociation of RAS protein from GTP, which in turn activates the MAPK pathway and exerts carcinogenic effects. When PTEN was mutated, the PI3K pathway was continuously activated and exerts carcinogenic effects. PINK1 exerts a carcinogenic effect by activating AKT, thereby activating the downstream signaling pathway. LKB1 cannot activate AMPK when it is mutated, and cannot negatively regulate the mTOR pathway, which, like RICTOR amplification, causes excessive activation of the mTOR pathway and plays a carcinogenic role.

Although the abovementioned gene targets have been found to be carcinogenic drivers in NSCLC and because targeted therapy has greatly ameliorated prognosis, the majority of patients with advanced non-small cell lung cancer lack known oncogenic drivers, and no targeted therapy has been developed for these patients. Studying the pathogenesis of non-small cell lung cancer, we observed that overactivation of the MAPK pathway and PI3K/AKT pathway is a cause of NSCLC formation (Figure 2), and abnormal activation of these pathways is also an important drug-resistance mechanism in NSCLC. The focus has been directed to the study of drugs targeting the cancer-causing pathway for the treatment of NSCLC. The NSCLC targets discussed in this section were discovered in recent years. Research on these targets will assist to forward understand the occurrence and development of NSCLC and will also facilitate researchers develop drugs against these targets in the future.

### NGRs

NRGs (neuregulins) constitute an intricate family of structurally related cellular growth factors including NRG1, NRG2, NRG3, NRG4, NRG5 and NRG6, which participated primarily in the growth of the nervous and cardiovascular systems (Nagasaka and Ou, 2022). Members of the NRGs family all have an epidermal growth factor (EGF)-like domain of approximately 65 amino acids, the domain is critical for NRGs attaching to the ErbB receptor tyrosine kinase (RTK) family members (EGFR, ErbB2, ErbB3, and ErbB4) (Nagasaka and Ou, 2022). These RTKs consist of a C-terminal tail, a kinase domain, a transmembrane domain, a short intracellular juxtamembrane domain, and a large extracellular ligand-binding domain. These transmembrane receptors engage ligands and then form hetero- or homodimers. This causes the phosphorylation of their intrinsic kinase domain, which activates the PI3K-AKT and MAPK pathways downstream (Trombetta et al., 2021). Non-autonomous receptors ErbB2 and ErbB3, whose activation is reliant on heterodimerization with other ErbB receptors, ErbB2 is the preferred dimerization partner of the other three ErbB RTK-family receptors but is unable to bind with growth factor ligands. In general, ErbB 3 is thought to be kinase lacking (Trombetta et al., 2021). Under normal circumstances, NRG1 binds primarily to ErbB 3 and forms heterodimers of ErbB2/ErbB3 to activate downstream signaling pathways (Laskin et al., 2020). NRG2 binds primarily to ErbB4 and forms ErbB 4 homodimers to activate downstream signaling pathways (Falls, 2003; Nagasaka and Ou, 2022).

NRG1 fusions cause the abnormal production of the EGF-like domain of NRG1 on the cellular membrane, which results in the pathological activation of the PI3K/AKT, MAPK, and other signaling pathways, thereby resulting in abnormal cell proliferation (Figure 2) (Laskin et al., 2020). Shin et al. demonstrated that enhanced phosphorylation of FAK and Src by an SLC3A2-NRG1 fusion caused cancer cell migration. NRG1 fusion partners that have been found in lung cancer thus far include CD74, SLC3A2, WRN, VAMP2, RALGAPA1, TNC, MRPL13, DPYSL2, FGFR1 PARP8, CADM1 F11R, MDK, DIP2B,FLYWCH1, KRAS, ATP1B1, ROCK1, PLCG2, SDC4, RBPMS, VAPB, and ITGB1 (Laskin et al., 2020). NRG1 fusion accounts for approximately 1%–2% of NSCLC cases. There is evidence that NRG1 fusion proteins are more common in women and non-smokers (Nakaoku et al., 2014; Laskin et al., 2020). Besides, patients with other solid tumors, like pancreatic ductal adenocarcinoma (PDAC), are also shown to have NRG1 fusion (Nagasaka and Ou, 2022).

More recently, in a group of patients with lung adenocarcinoma (LUAD) without a known carcinogenic driver, an NRG2 fusion protein (CD74-NRG2α) was discovered, and the CDH1-NRG2α fusion protein and F11R-NRG2α chimeric transcript were subsequently discovered (Kohsaka et al., 2020; Ou et al., 2021b). Tumor cells with these fusion proteins do not express ErbB3 but do express ErbB4 (Trombetta et al., 2021). NRG2 fusion, by binding to ErbB4, leads to the activation of downstream signaling pathways, exerting a carcinogenic effect, and this pathway needs to be further explored.

NRG1 fusion proteins are also closely related to cancer drug resistance. Immature progenitor cells can acquire characteristics of cancer stem cells thanks to the CD74-NRG1 fusion, enabling them to resist chemotherapy and targeted therapies (Laskin et al., 2020; Trombetta et al., 2021). Moreover, activation of the pathway caused by NRG1 fusion may act as a mechanism of resistance to specific TKIs and ALK inhibitors (Cadranel et al., 2021; Trombetta et al., 2021). Studies have showed that NSCLC cells directly activate ErbB pathways by activating the NRG1-ErbB3-EGFR axis when treated with second-generation ALK inhibitors (Trombetta et al., 2017). An NRG1 fusion protein in conjunction with ALK fusion proteins was found in sample taken by rebiopsy of ALK-positive patients treated with alectinib (Trombetta et al., 2021).

The prognosis for lung cancer patients with the NRG1 fusion protein is not promising. NRG1 fusions had a higher probability of extrathoracic metastasis and a bigger tumor, and chemotherapy and immunotherapy do not show effectiveness (Trombetta et al., 2021). Blocking the activity of the NRG1-ErbB3 pathway is a reasonable method to treat tumors with an NRG1 fusion protein. Theoretically, NRG1 fusion or NRG2 fusion malignancies can be treated by inhibiting the NRG/ErbB cascade using ligand receptor-binding inhibitors or ErbB antidimerizing drugs. Zenocutuzumab is a new type of bispecific anti-ErbB2/anti-ErbB3 antibody in the IgG1 class that disturbs the combination of NRG1 and ErbB3 and restrains the formation of ErbB2/ErbB3 heterodimers. In 2021, due to the initial efficacy of zenocutuzumab in clinical trials, the FDA approved zenocutuzumab for the treatment of NRG1-fusion cancers (Schram et al., 2022). Seribantumab is a monoclonal IgG2 antibody against ErbB3, and it has also been shown to be a potentially beneficial treatment for NRG1-fusion cancers (Laskin et al., 2020; Nagasaka and Ou, 2022; Schram et al., 2022). A humanized monoclonal antibody with a strong affinity for ErbB3 Domain III is GSK2849330, blocking ErbB3 binding with NRG1 and thus inhibiting receptor heterodimerization (Ke et al., 2019; Laskin et al., 2020; Trombetta et al., 2021; Schram et al., 2022). In a recurrent, unresectable late invasive mucinous adenocarcinoma patient carrying a CD74–NRG1 fusion, 1 year and 7 months was the confirmed duration of a durable partial response (Trombetta et al., 2021). Many other drugs targeting this route are also being tested. Although only the fusion of NRG1 and NRG2 has been found in tumors, other members of the NRG family are being evaluated because they have an EGF-like domain that can bind to the ErbB RTK receptor and disrupt signaling.

### CLIP1-LTK

Recently, it has been reported that CLIP1-LTK fusion gene is a new carcinogenic driver in NSCLC and is present in 0.4% of NSCLC cases (Izumi et al., 2021). CLIP1 (CAP-Gly domain-containing linker protein 1) belongs to the family of microtubule plus-end tracking proteins. As a member of the ALK/LTK subfamily of receptor tyrosine kinases, LTK (leukocyte receptor tyrosine kinase) is similar to ALK, sharing more than 80% of the kinase domain (Izumi et al., 2021; Cooper et al., 2022). The RAS/MAPK and PI3K/AKT signaling pathways can both be activated by LTK (Figure 2) (Cooper et al., 2022). Due to its kinase activity, the CLIP1-LTK fusion causes oncogenic transformation in NSCLC patients (Izumi et al., 2021). A greater risk of metastasis has been linked to early-stage NSCLC with high LTK expression (Cooper et al., 2022). At present, the function of LTK is not clear (Cooper et al., 2022). Inhibitors targeting LTK are lacking. Lorlatinib, an ALK inhibitor, can decrease CLIP1-LTK kinase activity since the kinase domains of LTK and ALK are highly similar, showing therapeutic effects on NSCLC patients with *CLIP1-LTK* fusion gene. The *CLIP1-LTK* fusion gene may become a candidate target for certain ALK inhibitor therapy, such as lorlatinib (Izumi et al., 2021). Selective LTK-TKIs need to be developed for clinical treatment.

### DDR

Discoidin domain receptors (DDRs) constitute a distinct subclass of tyrosine kinase superfamily of transmembrane receptor; this subfamily consists of DDR1 and DDR2, which play a key role in regulating basic cell processes, such as proliferation, migration, invasion, morphogenesis, and adhesion (Figure 2) (Kothiwale et al., 2015; Elkamhawy et al., 2021; Gao et al., 2021). Collagen can attach to DDR and trigger gradual tyrosine autophosphorylation, which in turn activates DDR signaling (Elkamhawy et al., 2021; Gao et al., 2021; Zhu et al., 2022). Numerous illnesses, including NSCLC, ovarian cancer, breast cancer, and various inflammatory and neurodegenerative conditions, are linked to DDR dysregulation (Elkamhawy et al., 2021). In NSCLC, high DDR1 expression has been linked to a poor prognosis. DDR1 overexpression has been strongly linked to lymph node metastases in NSCLC patients (Elkamhawy et al., 2021). DDR1 positivity was detected in as many as 61% of the 171 cases of aggressive NSCLC that were subjects of an immunohistochemical examination (Gao et al., 2021). Lung cancer cells’ bone metastases are significantly influenced by DDR1 (Valencia et al., 2012). One study showed that downregulating DDR1 inhibited the migration and invasion of melanoma cells (Elkamhawy et al., 2021). A 3%–4% prevalence of lung squamous cell carcinoma (LUSC) patients have DDR2 mutations, and it is possible that these mutants coexist with other carcinogenic drivers, such as KRAS G12C (Nicos et al., 2014; Rammal et al., 2016). With a Tp53^L/L^ mouse model, a study reported the discovery that lung cancer cells with the L63V mutation in DDR2 are poorly differentiated (Gao et al., 2021). The DDR2 gene has been linked to mutations in a number of tumor types, including head and neck carcinoma, colorectal carcinoma, bladder cancer, melanoma, gastric cancer, and cervical carcinoma (Rammal et al., 2016). Scientists have concluded that DDR1 is a major contributor to chemotherapy drug resistance in tumor cells; the activation of NF-κB and its related effectors is assumed to be the mechanism by which this resistance is achieved, thus hindering chemotherapy-induced apoptosis (Rammal et al., 2016). Strategies to inhibit mutant DDR effects in cancer are promising. A number of BCR-ABL inhibitors have been found to inhibit DDR1 and DDR2. Dasatinib administered at very low concentrations inhibits DDR1 activity (Elkamhawy et al., 2021). Another study on lung cancer cells carrying “gain-of-function” DDR2 mutations reported that dasatinib demonstrated a very promising therapeutic effect (Elkamhawy et al., 2021). XBLJ-13 is a highly specific and effective DDR inhibitor, which potently represses DDR1 and DDR2 kinases and shows a good pharmacokinetic profile and *in vivo* efficacy (Dong et al., 2022). Recently, it was discovered that the universal Type II kinase inhibitor DDR1-IN-1, a specific DDR1 inhibitor, binds to DDR1 in the DFG-out conformation to prevent DDR1 autophosphorylation in cells at submicromolar doses (Kothiwale et al., 2015; Elkamhawy et al., 2021). Another recently reported DDR1 inhibitor is compound KST9046 (Elkamhawy et al., 2021). Moreover, an anti-DDR mAb is also in development and shows high specificity. Specifically, Human glioma cell G140 invasion and adhesion are inhibited by DDR1 mAb 48B3. In 2019, Tao et al. created a novel antibody-drug conjugate named T4H11-DM4, a drug including the anti-DDR1 antibody and DM4 (a tubulin inhibitor that prevents cell proliferation), which inhibited colon cancer growth both *in vitro* and *in vivo* (Tao et al., 2019; Gao et al., 2021).

### KRAS G12C

KRAS (Kirsten rat sarcoma viral oncogene homolog) belongs to the RAS gene family. It is crucial for the signaling system that promotes the proliferation of tumor cells as well as angiogenesis, involving, for example, RAS-RAF-MEK-ERK pathway and PI3K-AKT-mTOR pathway (Figure 2) (Veluswamy et al., 2021). The KRAS gene is permanently active and unable to create regular RAS proteins when it has been altered, then disrupting intracellular signaling, causing uncontrolled cell proliferation and ultimately inducing cancer. One of the most frequent oncogenic drivers in advanced non-small cell lung cancer is mutant KRAS, and typically, it is expressed in a mutually exclusive manner; that is, it is not expressed with other clinically relevant driver mutants, like EGFR, BRAF or ALK mutation, although it is frequently comutated with the tumor suppressor genes STK11, TP53, and CDKN2A/CDKN2B(Davis et al., 2021; Veluswamy et al., 2021; Punekar et al., 2022). KRAS gene mutations are seen in 25% of NSCLCs, of which LUAD accounts for 30%–50%. At codon 12, where the glycine residue is replaced by other amino acids, more than 80% of oncogenic KRAS mutations take place, resulting in the genomic heterogeneity of KRAS-mutant tumors (Veluswamy et al., 2021; Punekar et al., 2022). In NSCLC, approximately 44% of KRAS mutations are glycine-to-cysteine mutations (G12C), and 13% of all LUAD patients had the KRAS G12C mutation. Moreover, KRAS G12C is present in 3% of cases with colorectal cancer (CRC) and in 1% of cases with pancreatic ductal adenocarcinomas (PDACs) and other solid cancers (Veluswamy et al., 2021). KRAS G12C is strongly associated with smoking and is more common in smokers (Veluswamy et al., 2021).

Drugs targeting the KRAS G12C mutant are in clinical trials, and sotorasib is already on the market. Sotorasib is a small molecule designed to bind to KRAS G12C and it stops the protein from transmitting signals that promote unrestrained cell proliferation by locking it in an inactive state (Parums, 2022). The method by which the drug is targeted does not affect the unmutated KRAS protein. In 124 patients with advanced-stage KRAS G12C-mutant NSCLC who had formerly received chemotherapy and/or immunotherapy, sotorasib demonstrated a 37.1% objective response rate (ORR), an 11.1-month median duration of response (DOR), a 6.8-month median PFS, and a 12.5-month median overall survival (OS) in the Phase I/II CodeBreaK 100 clinical study (Punekar et al., 2022). Subsequently, For the treatment of individuals with locally advanced or metastatic NSCLC that has the KRAS G12C mutation, the FDA authorized sotorasib in 2021 (Punekar et al., 2022). Adagrasib is another potentially marketable KRAS G12C inhibitor. On 16 February 2022, the U.S. FDA accepted a new drug-marketing application for Adagrasib (MRTX849) used in the treatment for NSCLCs with the KRAS G12C mutation. Comments have not yet been given. According to the data from phase II KRYSTAL-1 trial, among 116 NSCLCs who had previously received treatment, the ORR of adagrasib was 42.9%, the disease control rate (DCR) was 79.5%, the median DOR was 8.5 months, the median PFS was 6.5 months, the median OS was 12.6 months, and the estimated 1-year OS was 50.8% (Punekar et al., 2022). Additionally, adagrasib showed a therapeutic effect on NSCLC with intracranial metastasis (Punekar et al., 2022). With promising preliminary results from ongoing Phase Ib/II clinical trials, JDQ443 is a covalent KRAS G12C inhibitor that is now undergoing clinical development (Lorthiois et al., 2022). Several other KRAS G12C inhibitors are in clinical trials and are expected to show positive therapeutic outcomes.

### RASGRF1

RAS protein-specific guanine nucleotide-releasing factor 1 (RASGRF1), is a kind of RAS guanine nucleotide exchange factor (RASGEF), specifically induces GDP/GTP exchange with many members of the RAS GTP enzyme family, including H-RAS, N-RAS and KRAS (Tarnowski et al., 2012; Pan et al., 2021). RAS exists in two conformations: GDP-bound, the inactive form, and GTP-bound, which initiates a sequence of molecular events through signaling to downstream effectors (Cooper et al., 2020). RASGRF1 promotes dissociation of GDP from a RAS protein, allowing GTP to bind and thus activate a downstream signal cascade (Tarnowski et al., 2012; Cooper et al., 2020). RASGRF1 has been proved to be differentially expressed in diverse types of tumors. Recent studies have demonstrated that certain RASGRF1 fusion are oncogenic drivers in NSCLC that primarily affect the MAPK and PI3K signaling pathways; these include the TMEM87A-RASGRF1 fusion and OCLN-RASGRF1 fusion (Cooper et al., 2020; Hunihan et al., 2022). Similarly, similar fusions have been detected in other types of tumors; for instance, the SLC4A4-RASGRF1 fusion has been found in pancreatic ductal adenocarcinoma, IQGAP1-RASGRF1 fusion in sarcoma, TMEM154-RASGRF1 fusion in acute myeloid leukemia, CD63-RASGRF1 and EHBP1-RASGRF1 fusion in melanocytic neoplasm and ABCC2-RASGRF1 fusion in melanoma (Hunihan et al., 2022). It has been reported that the pleckstrin homology (PH) 1 domain of RASGRF1 negatively regulated GEF activity, while PH2 domain is necessary for RASGRF1 to induce ERK activity (Cooper et al., 2020). The entire catalytic domain of RASGRF1 is preserved, and the regulatory domain is functionally impaired in all of the aforementioned fusion. The regulatory PH1 domain is absent from the TMEM87A-RASGRF1 fusion, and the PH2 domain that activates ERK is still present in this fusion. Losing its self-inhibitory region of the RASGRF1 gene leads to continuously activated RAS-GTP and downstream signaling pathways, promoting cell transformation and tumor formation (Figure 2) (Cooper et al., 2020). Moreover, the TMEM87A-RASGRF1 fusion may mediate drug resistance. The proliferation of the PC9 human EGFR-mutant lung cancer cell line completely depends on EGFR signaling, and EGFR tyrosine kinase inhibitors are able to stop it. PC9 cells carrying the TMEM87A-RASGRF1 fusion, however, showed resistance to tyrosine kinase inhibitors. The therapeutic effect of MAPK pathway inhibitors was more effective on this mutant cell line (Cooper et al., 2020). The inhibition of the RAF-MEK-ERK pathway in RASGRF1-fusion tumors is likely a potential therapeutic target. Additionally, RASGRF2 fusion proteins have recently been reported in melanocytic lesions (Houlier et al., 2021).

### PTEN

PTEN (phosphatase and tensin homolog), which was recently identified as a tumor suppressor gene with bispecific phosphatase activity, is also a gene that is second only to the P53 gene in its close relationship to carcinogenesis and plays an important role in cell proliferation, apoptosis, adhesion, migration, and infiltration (Liu et al., 2022; Fang et al., 2022). PTEN is frequently inactivated in various malignancies, such as brain, prostate, endometrial, gastric cancers and NSCLC, and clinical data have suggested that PTEN loss of function occurs in 10%–25% of NSCLC cases (Liu et al., 2022). To date, studies have indicated that PTEN loss of function mainly affects the PI3K/AKT pathway, thereby affecting cell proliferation, migration and other processes (Figure 2) (Cho et al., 2022). Under normal circumstances, the PI3K signaling pathway is activated by cell surface-located growth factor receptors, cytokine receptors, GPCRs (G protein-coupled receptors), and integrins. Then, phosphatidylinositol 4,5-bisphosphate (PIP2) was phosphorylated by PI3K, turning it to phosphatidylinositol-3,4,5-triphosphate (PIP3), a critical factor in activating downstream AKT, thereby mediating succeeding signals including cell survival, proliferation, and migration. Dephosphorylating PIP3 to produce PIP2 then antagonizing PI3K and stopping the activation of AKT are all effects of PTEN-lipid-phosphatase activity (Liu et al., 2022). PTEN loss of function results in overactivation of the PI3K signaling pathway, which has been linked to the cancer pathogenesis. Furthermore, evidence suggests that the activation of PI3K/AKT signaling pathway contributes to cancer therapeutic resistance, including resistance to conventional chemotherapy, immunotherapy and agents targeting other oncogenic drivers. Drug resistance may be efficiently reduced by inhibiting PI3K/AKT signaling (Fang et al., 2022). PTEN can also affect tumor proliferation and migration through other pathways. A recent study reported that PTEN inhibited AMPK phosphorylation and activity. PTEN loss increased the general cell migration rate *via* its upregulation of AMPK activity, which enhanced energy production and sustained the cell motility machinery by controlling the polarized trafficking of mitochondria (Peglion et al., 2022). Emerging evidence suggests that PTEN loss is correlated with immunotherapy resistance. In BRAF mutant melanoma cells, PTEN loss negatively affects anti-tumor immunity and T cell tumor recruitment. Secondly, in melanoma patients, clinical data show that the decrease of PTEN expression is related to the drug resistance of anti-PD1 therapy (Fruman et al., 2017). In addition, in melanoma patients with heterogeneous PTEN expression regions, T cell infiltration in sub-regions lacking PTEN protein expression is always low (Fruman et al., 2017). To date, no drugs target PTEN. However, a PTEN gene nanovector (NP-PTEN), created using branch-PCR, showed excessive PTEN protein abundance, which restored PTEN function by deactivating the PI3K-AKT-mTOR signaling cascade, thereby inhibiting cell growth and inducing apoptosis. The mean tumor volume and tumor weight were decreased by 61.7% and 63.9%, respectively, in mice with NCI-H1299 tumor xenografts when intratumorally injected NP-PTEN as compared to control mice (Lu et al., 2022). Thus, targeting PTEN for cancer treatment is a promising research direction. In the future, we need to conduct more in-depth research on this topic due to the prevalence of PTEN inactivation in tumors, which not only will contribute to the treatment of NSCLC but may also benefit the treatment of other tumors.

### STK11/LKB1

In the 1990s, STK11 (serine threonine kinase 11) was identified as a significant tumor suppressor gene (Sumbly and Landry, 2022). Clinical data show that a wide variety of tumors carry STK11/LKB1 abnormalities (Sumbly and Landry, 2022). STK11 encodes liver kinase B1 (LKB1), a protein kinase important in maintaining cellular energy balance (Sumbly and Landry, 2022). LKB1 can phosphorylate AMP-activated protein kinase (AMPK). Normal cells activate AMPK when LKB1 is present, inhibiting tumor development and lengthening survival (Sumbly and Landry, 2022). STK11/LKB1 mutations cause the partial loss of AMPK regulation, subsequently resulting in abnormal mTOR and HIF-1-α expression patterns (Figure 2) (Mograbi et al., 2021; Sumbly and Landry, 2022). In NSCLC, STK11/LKB1 mutations routinely occur, with an incidence of approximately 6%–13.6% (Shire et al., 2020), and approximately 7% of its mutation coexists with a KRAS mutation (Sumbly and Landry, 2022). NSCLC patients carrying STK11/LKB1 mutations are more likely to have an inferior prognosis than those withoutSTK11/LKB1 mutation, and specifically, STK11/LKB1 mutations have been closely associated with poor prognosis in NSCLC (Rosellini et al., 2022; Sumbly and Landry, 2022). The downregulation of STK11/LKB1 appears to promote carcinogenesis and metastasis *via* boosting the expression of proangiogenic genes and epithelial-mesenchymal transition (EMT) inducers. Moreover, STK11/LKB1 mutations have been found to be more likely connected with an immunosuppressive microenvironment. Mutations in STK11 confer resistance to immunotherapy due to downregulating the expression of programmed death ligand 1 (PD-L1) (Rosellini et al., 2022; Sumbly and Landry, 2022). According to one study, LKB1 deletion affects radiation resistance *via* activating the KEAP1/NRF2 pathway and upstream NRF2 synthesis and reducing reactive oxygen species (ROS) levels (Sumbly and Landry, 2022). Treatment for STK11-mutated NSCLC is challenging, and research on STK11/LKB1 mutations as targets for NSCLC treatment deserves to be pushed forward. Data from a recent clinical trial suggested that inhibitors of mTOR and glutamine, such as everolimus and telaglenastat, exerted a therapeutic effect on NSCLC patients with STK11/LKB1 mutations (Ndembe et al., 2022; Sumbly and Landry, 2022).

### PELP1

**FIGURE 3:**
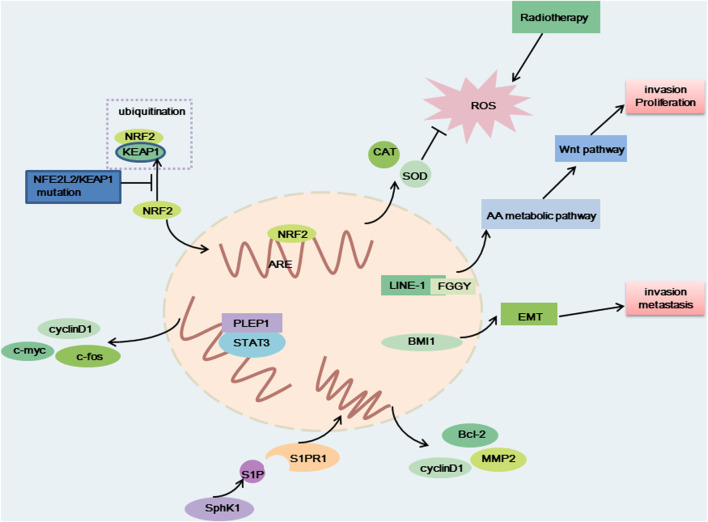
The carcinogenic effect of NFE2L2/KEAP1 mutation, PELP1, LINE-1-FGGY, SphK1 and BMI1. When NFE2L2/KEAP1 was mutated, NRF2 cannot be degraded, and NRF2 binds to antioxidative response element (ARE) to produce antioxidant factors that resist the therapeutic effects of radiotherapy. LINE-1-FGGY promotes cell proliferation and invasion by influencing the AA metabolic pathway as well as the Wnt pathway. BMI1 promotes cell transfer and invasion by influencing EMT. The binding of PELP1 to STAT3 promotes the expression of c-myc, cyclin D1, c-fos, and thus promotes cell proliferation. Elevated SphK1 activity promotes S1P production, which binds to corresponding receptors and promotes the production of Bcl-2, MMP2 and cyclin D, thereby promoting cell proliferation and metastasis.

PELP1 is a scaffolding protein called proline, glutamate, and leucine-rich protein 1 that is an important coregulator of multiple transcription factors and nuclear receptors (Slowikowski et al., 2015; Wang et al., 2022). Recognized as a proto-oncogene, *PELP1* contributes significantly to carcinogenesis and the growth of numerous cell lines. The regulation of several crucial processes, such as estrogen signaling, cell cycle progression, ribosome synthesis, and the DNA damage response, is aided by PELP1 (Wang et al., 2022). PELP1/STAT3 complexes enhanced the expression of c-myc, cyclin D1, and c-fos in a c-Src-and MAPK-dependent way upon stimulation (Figure 3) (Vadlamudi et al., 2005). Moreover, a series of clinical data showed that its expression in cancer tissue differs from that in normal tissue, like breast, prostate, lung, and ovary cancers (Girard et al., 2014; Wang et al., 2022). In most malignancies, PELP1 overexpression has been linked to a worse prognosis and a larger tumor grade (Girard et al., 2014; Wang et al., 2022). According to a study, node-positive and metastatic breast cancers express PELP1 two to three times more than node-negative breast tumors do (Wang et al., 2022). The mRNA and protein levels of PELP1 are increased in the context of NSCLC in comparison to those in nearby normal lung tissue (Slowikowski et al., 2015; Wang et al., 2022). Dysregulation of PELP1 boosts MAPK signaling and has an impact on genes involved in cell proliferation, which aids in the proliferation of lung cancer cells (Wang et al., 2022). Inhibiting PELP1 prevented lung cancer cells from proliferating, forming colonies, migrating, and invading. Moreover, PELP1 has been associated with resistance to TKIs in NSCLC. Gefitinib sensitivity was boosted in lung cancer cells by PELP1 inactivation. In addition, gefitinib’s inhibition of EGFR signaling decreased the expression of the PELP1 protein, whereas EGF’s stimulation of the EGFR pathway increased PELP1’s protein expression in lung cancer cells (Wang et al., 2022). These findings provide new ideas, and for NSCLC treatment options, the joint application of PELP inhibitors and TKIs is worth exploring.

### NFE2L2/KEAP1

Nuclear factor erythroid-2-related factor-2 (NFE2L2) encodes a crucial transcription factor named NRF2, playing an important role in the cellular antioxidant response (Xu et al., 2020; Ji et al., 2022). Evidence indicated that the transcription of cytokines, chemokines, and type I interferon-inducing cGAS/STING signaling were interfered with by NRF2 (Scalera et al., 2022). KEAP1 (kelch-like ECH-associated protein 1) can degrade NRF2 *via* the KEAP1-CUL3-RBX1 E3 ubiquitin ligase complex (Figure 3) (Xu et al., 2020). A key regulator of cellular homeostasis that enables cells to withstand oxidative and metabolic stressors is the KEAP1-NRF2 axis (Scalera et al., 2022). KEAP1 is regarded as a tumor-suppressive gene, and research has shown that a KEAP1 loss-of-function mutation promotes tumor growth (Scalera et al., 2022). The majority of the oncogenic NFE2L2 mutations are found at KEAP1-binding sites and prevent KEAP1 from degrading NFR2, which results in constitutive activation of NRF2-driven gene transcription (Scalera et al., 2022). Previous research has demonstrated that the R34P, R34G, and R34Q mutations cause the production of mutant versions of NRF2 that are not targets for ubiquitination and destruction because they are not substrates for KEAP1 (Cannataro et al., 2022).

Mutations in the KEAP1-NRF2 pathway are common in NSCLC. 20% of patients with LUAD and 25%–30% of patients with LUSC have KEAP1 and NFE2L2 mutations, respectively (Scalera et al., 2022). Lung tumors with NFE2L2 mutations have typically had a bad prognosis (Cannataro et al., 2022). Preclinical and clinical studies in NSCLC have suggested that KEAP1 and NFE2L2 mutations confer resistance to chemotherapy, radiotherapy, and targeted agents (Ji et al., 2022; Scalera et al., 2022). Specifically in lung cancer, NRF2 dysregulation results in resistance to EGFR tyrosine kinase inhibitors and a number of inhibitors that target the RTK/RAS/MAPK pathway (Scalera et al., 2022). Contrarily, immunotherapy has been observed to improve prognosis and survival in NSCLC patients who carry the NFE2L2 mutation, which may be attributable to the correlation between KEAP1 and NFE2L2 mutations and elevated expression of PD-L1 (Xu et al., 2020). Few NFE2L2 targeted treatments have been created. Nevertheless, the result of one Phase II trial on advanced NSCLC harboring NRF2-activating alterations gives us hope. TAK-228 (a TORC1/2 inhibitor) achieved a good therapeutic response in LUSC patients with NFE2L2 mutations, with a 25% overall response rate and a 8.9 months median PFS (Paik et al., 2022). One potentially promising therapeutic strategy is the selective degradation of mutant forms of NRF2. Direct NRF2 inhibition-based therapies should be aggressively pursued as they may more effectively shut down the various carcinogenic pathways induced by abnormal NRF2 activity. ML385, a promising NRF2-specific inhibitor, can specifically bind to the DNA-binding domain of the transcription factor NRF2, block its interaction with the promoter region of a downstream target genes and inhibit the transcription and expression of the target gene. Moreover, ML385 exerted a more significant antitumor effect when used in combination with chemotherapy drugs such as carboplatin, and lung cancer cells’ sensitivity to chemotherapeutic medicines was significantly increased by ML385 (Ji et al., 2022).

### RICTOR

Rapamycin insensitive companion of mTOR (RICTOR), an mTORC2-specific cofactor, is an upstream kinases of several AGC kinases, such as AKT (Cheng et al., 2015). One of the two distinct mTOR complexes, mTORC2, senses environmental stimuli and controls numerous cellular functions, such as cell development, proliferation, and metabolism, all of which, when improperly controlled, promote cancer (Figure 2) (Kim et al., 2020). Under the background of these different functions, mTORC2 plays a key carcinogenic role in regulating the migration, invasion and metastasis of breast cancer, ovarian cancer, prostate cancer, colorectal cancer and glioma (Cheng et al., 2015). Furthermore, animal experiments have suggested that overexpression of RICTOR induced malignant glioma formation in a transgenic mouse model (Cheng et al., 2015). Clinical research has indicated that RICTOR amplification is the only tumor-specific genetic change among the cancer-related genes inspected in an 18-year-old NSCLC patient without a history of smoking (Cheng et al., 2015). RICTOR amplification commonly occurs in NSCLC; it has been identified in roughly 10.3% of LUAD patients and 15.8% of LUSC patients (Cheng et al., 2015). The PI3K/AKT/mTOR pathway’s other genes were altered in one-third of the patients with RICTOR amplification (Cheng et al., 2015). Selective RICTOR or mTOR2 inhibitors have not been developed, but mTOR1/2 inhibitors have shown good therapeutic activity in RICTOR-amplified lung cancer cells. After receiving treatment with dual mTOR1/2 inhibitors, such as the recently developed MLN0128 drug and the previously developed CC-223 drug, the tumor was stable for more than 18 months (Cheng et al., 2015).

### PINK1

Phosphatase and tensin homolog-induced kinase 1(PINK1) is a 581-amino acid protein with a highly conserved serine/threonine protein kinase domain, in addition, possessing a mitochondrion-targeting motif and a regulatory C-terminal sequence (Zhang et al., 2017). The dynamics of mitochondrial homeostasis, including mitophagy, fission, and fusion, are influenced by PINK1 (Zhang et al., 2017; Chang et al., 2018). PINK1 upregulation correlated with overexpression of the principal tumor suppressor PTEN (Zhang et al., 2017). Over the past 10 years, research has shown that PINK1 is crucial for cell survival and anti-apoptotic actions and the cell cycle *via* its mechanistic effects mediated *via* proteasomal, autophagic, PI3K/AKT, and NF-κB pathways and calcium-dependent signaling. As a tumor oncogene, PINK1 is closely related to the main oncogenic PI3K/AKT axis (Zhang et al., 2017). Recent studies have shown that PINK1 activates AKT through the mTORC2/mitochondrial control axis to augment the aggressiveness of cancerous cell and accelerate the renewal of cancer stem cells through Notch signaling (Figure 2) (Zhang et al., 2017). PINK1 is upregulated in breast, colorectal and endometrial cancer and NSCLC (Zhang et al., 2017; Lu et al., 2020). High PINK1 expression has been identified as a poor prognostic factor for LUAD (Chang et al., 2018). PINK1 overexpression promoted the proliferation of non-small cell lung cancer cells. PINK1 knockdown resulted in a decrease in the proliferation rate of NSCLC cells, a decrease in colony-forming capacity, and an increase in cell cycle arrest (Zhang et al., 2017). In addition, these findings suggest that PINK1 induces lung cancer cell resistance through the NF-κB pathway. When PINK1 binds to TRAF6 and TAK1, it promotes TRAF6’s autodimerization and autoubiquitination, which activates the NF-κB pathway (Zhang et al., 2017). Deletion of PINK1 may make breast cancer cells sensitive to paclitaxel and NSCLC cells sensitive to cisplatin, while PINK1 overexpression can counteract this sensitization and result in chemical resistance (Zhang et al., 2017). The mechanisms underlying PINK1 protection and resistance in cancer cells indicate that PINK1 is a target for cancer therapy and specifically for NSCLC. However, to clarify the precise function played by PINK1 in the etiology of NSCLC, more research is required.

### LINE-1

In the human genome, Long interspersed nuclear element-1 (LINE-1) is the most abounding (number of bases) known retrotransposon (approximately 500,000 copies), accounting for approximately 17% of the human genome sequence (Sun et al., 2022). LINE-1 is also the only known transposon in the human genome that can undergo spontaneous transposition. As a widely distributed transposable element, LINE-1 promotes tumor growth by interfering with the transcription of genes relevant to tumors and triggering genome instability (Sun et al., 2022). Through somatic LINE-1 retrotransposition (LRT) or activation of the LINE-1 antisense promoter (LINE-1-ASP), transcriptionally active LINE-1 creates a LINE-1-gene chimeric transcript (LCT) that functions as an oncogene in the development of cancer (Sun et al., 2022). Genome instability, increased metabolic activity, decreased immunological responses, and a particular clinical condition have all been linked to LCT activity (Sun et al., 2022). LCTs were reported to have abnormally high expression and to be linked to carcinogenic activity through LINE-1-ASP activation in the majority of cancer tissues. Increased LINE-1 activity has been directly linked to a number of illnesses, including cancer and neurological degenerative conditions. It has been reported that >50% of NSCLC patients exhibit increased LINE-1 ORF1 protein expression, and ORF1 is a protein involved in ribonucleoprotein assembly, chromatin remodeling and altered gene expression (Reyes-Reyes et al., 2017). Studies have shown that LINE-1 activity in NSCLC was higher than that in normal tissues and affected mitochondrial function and metabolic processes. Five LCTs (LINE-1-MCM3, LINE-1-PHF20, LINE-1-INPP4B, LINE-1-SLC44A5, and LINE-1-SUGCT) were detected in both LUAD and LUSC samples, indicating that they may be involved in the tumorigenesis of NSCLC. What’s interesting is that INPP4B and SUGCT have been related with metabolic pathway or metabolic disorder. There have been earlier reports linking MCM3 and INPP4B to cancer (Sun et al., 2022). Moreover, LINE-1 reactivation has been linked to poor prognosis in NSCLC (Bojang and Ramos, 2018).

FGGY, a metabolic gene that encodes carbohydrate kinase, is usually regarded as a tumor suppressor gene involved in arachidonic acid (AA) metabolism (Sun et al., 2022). The metabolites of AA, an important fatty acid, take part in a number of physiological processes in cells, including cell division and migration. One of the most noticeable tumor-specific LCTs in LUSC, LINE-1-FGGY is produced by the LINE-1-ASP activation-mediated transcription of the intron region in LINE-1 through exon 13 in FGGY (Figure 3) (Sun et al., 2022). The oncogenic effects exhibited by LINE-1-FGGY were partially reversed by ML355, a metabolism inhibitor (Sun et al., 2022).

### SphK1

Sphingosine kinase 1 (SphK1) is a key enzyme in sphingomyelin metabolism that catalyzes sphingosine to produce sphingosine 1-phosphate (S1P) and is related to cell proliferation, metastasis, migration, as well as the epithelial-to-mesenchymal transition (EMT) (Ma et al., 2021). SphK1 and S1P signaling plays important roles in multiple diseases, such as cancer, diabetes and inflammation-related diseases, rheumatoid arthritis, atherosclerosis and multiple sclerosis. It was reported that SphK1 was overexpressed in many tumors, for example, prostate and breast cancers (Ma et al., 2021). Furthermore, overexpression of SphK1 has poor prognosis in patients with cancer. For instance, the overexpression of SphK1 has been significantly related to shorter survival time in patients with metastatic melanoma (Ma et al., 2021). There is evidence indicating that SphK1 expression is increased in NSCLC tissues and lung cancer cell lines. SphK1 facilitates the metastasis and proliferation of NSCLC cells (Ma et al., 2021). It was reported that STAT3 was a key gene to promote cell proliferation, and inhibit cellular immunity and apoptosis. S1P, the product of SphK1, functions after binding to corresponding receptors. There are five specific G protein coupled receptors, named, S1P receptor (S1PR)1–5. The continuous activation of STAT3 can promote activation of S1PR1. It was reported that SphK1 participates in the pathological process of NSCLC by regulating STAT3 (Ma et al., 2021). Moreover, it was demonstrated that SphK1 promotes the proliferation of NSCLC cells by regulating PI3K/Akt pathway. SphK1 activates the PI3K/AKT/NF-κB pathway and increases the expression of theapoptotic and migration -associated genes such as Bcl-2, MMP2 and cyclin D1 (Figure 3) (Song et al., 2011; Xue et al., 2022).

Research has suggested that SphK1 plays a role in chemotherapy resistance in breast, lung, colon, and hepatocellular cancer (Song et al., 2011). SphK1 facilitated autophagy and induced the EMT by promoting lysosomal degradation of CDH1/E-cadherin in hepatoma cells (Ma et al., 2021). Moreover, SphK1 increased radiochemotherapy drug resistance in breast cancer. It was reported that SphK1 could protect cancer cells from drug-induced apoptosisby increasing ceramide levels. Bonhoure and others reported that the ectopic overexpression of SphK1 was correlated with the resistance to doxorubicin and etoposidin HL-60 leukemia cells. The activation of SphK1 was associated with the resistance to docetaxel or camptothecin in prostate cancer cells (Song et al., 2011).

The antiapoptotic effect of SPHK1 on NSCLC cells has been associated with the activation of the NF-kB and PI3K/AKT pathways. Inhibition of SPHK1 may be a new way to treat NSCLC. SPHK1 plays an important antiapoptotic role in non-small cell lung cancer *in vivo*. Expressing or inhibiting the enzymatic activity of SPHK1 by silencing it may be a potentially effective strategy; therefore, SphK1 is an attractive drug target for developing anticancer therapies. The specific inhibitor SK1-I, either alone or in combination with chemotherapy, inhibited SPHK1 expression or SPHK1 activity, making NSCLC cells significantly more sensitive to apoptosis induced by chemotherapy drugs *in vitro* and *in vivo*. Injection of SK1-I effectively enhanced the tumor suppressive effect of docetaxel, a well-characterized clinically proapoptotic chemotherapy drug that targets the mammary gland, ovary and NSCLC tissues (Song et al., 2011). SKI-349 is a novel, highly efficient small-molecule SphK1/2 dual inhibitor. It has been shown to be not cytotoxic to human lung epithelial cells and to induce the apoptosis of NSCLC cells in experiments performed *in vitro* and *in vitro* (Xue et al., 2022).

### BMI1

B-cell-specific Moloney murine leukemia virus integration site 1 (BMI1) is an epigenetic regulator and an important component of Polycomb Repressive Complex 1, which regulates chromatin structures and thus the transcription of many important genes; it plays a significant part in regulating development, stem cell self-renewal, cell cycle, aging, cell differentiation, and tumorigenesis (Xiong et al., 2015; Shen et al., 2020). Initially, it was discovered to be a proto-oncogene that functions with c-myc to induce T-cell and B-cell lymphoma (Xiong et al., 2015). BMI1 has been found to cause tumorigenesis by modulating the transcriptional silencing of tumor suppressor genes such as p16^INK4a^, p19^ARF^, and p21^Cip1^ during cell senescence and proliferation or by inhibiting other tumor suppressor genes, such as PTEN, BCL2L11, and WWOX (Li et al., 2018). Amplification of the BMI1 gene or overexpression of BMI1-encoded proteins have been found in various cancer types. Abnormal overexpression of BMI1 has been found in gastric cancer, esophageal cancer, non-Hodgkin lymphoma, cervical carcinoma, breast cancer, colon carcinoma, melanoma, hepatocellular carcinoma, and NSCLC (Mu et al., 2016). In NSCLC, high BMI1 expression is a potent marker of poor prognosis and may be associated with BMI1 expression promoting the stemness properties of tumor cells (Koren et al., 2016; Shen et al., 2020). BMI1 overexpression has been tightly linked with cancer metastasis, invasion, and drug resistance. BMI1 overexpression can induce epithelial interstitial transformation and promote the occurrence of human LUSC and the invasion and metastasis of human LUSC cells by downregulating E-cadherin and upregulating waveform protein expression (Figure 3). Reducing BMI1 protein levels induced the apoptosis or aging of cancerous cells, increasing the sensitivity of cancerous cells to chemotherapy and radiotherapy (Shen et al., 2020). Inhibition of BMI1 in A549 NSCLC cells has been demonstrated to inhibit cell growth and significantly diminish A549 cell proliferation and tumorigenesis in nude mice (Xiong et al., 2015). BMI1 is a potential NSCLC therapeutic target and shows great potential to improve the therapeutic prospects for NSCLC. BMI1 inhibitors for cancer treatment are yet in the research and development phase. In 2014, the first BMI1 inhibitor, PTC-209, showed promising anticancer effects in preclinical models of several types of tumors. Another BMI1 inhibitor, PTC-596, which exhibits antileukemia activity *in vivo* and shows good safety, is in Phase 1 clinical trials (Shen et al., 2020).

### YES1

v-YES-1 Yamaguchi sarcoma viral oncogene homolog 1 (YES1) is a non-receptor tyrosine kinase belonging to the SRC kinase family (SFK) that exerts critical functional control of cell survival, proliferation, adhesion, migration, invasion, cell death, and angiogenesis and regulates a number of cancer signaling pathway (Figure 2) (Garmendia et al., 2022). YES1 amplification and overexpression are found in a variety of tumors, and siRNA- or shRNA-mediated YES1 downregulation can inhibit the proliferation and growth of rhabdomyosarcoma, NSCLC, and pancreatic cancer cell lines (Garmendia et al., 2022). YES1 amplification consists in 15% in LUAD and 25% in LUSC. High expression of YES1 in cancer has been closely associated with a poor prognosis (Hamanaka et al., 2019). Increased gene copy number or amplification of the YES1 gene has been found in recurrent NSCLC, esophageal squamous cell carcinoma, gastric cancer, and lymphoma (Garmendia et al., 2022). YES1 amplification and overexpression have also been found to be mechanisms for acquired resistance to different cancer treatments, as indicated by the inhibition of YES1 expression increasing cell sensitivity to chemotherapeutic drugs or targeted drugs, with dasatinib (an SFK inhibitor) treatment overcoming this drug resistance (Garmendia et al., 2019; Hamanaka et al., 2019; Garmendia et al., 2022). At present, SFK inhibitors (dasatinib, saracatinib and bosutinib) are mainly used in clinical cases of chronic myeloid leukemia (CML) and acute lymphoblastic leukemia (ALL) that are resistant or intolerant to imatinib (Garmendia et al., 2022). CH6953755 is a YES1-specific inhibitor that has exhibited selective and robust antitumor activity against YES1-amplified tumors *in vivo* and *in vitro* (Garmendia et al., 2022). Data on SFK inhibitors used in the clinical treatment of patients with NSCLC are lacking, but further investigation is warranted.

## Conclusion

Targeted therapy has greatly changed the prognosis of non-small cell lung cancer. However, many patients lack known carcinogenic drivers and thus do not benefit from the currently used targeted drugs. Common molecular pathological detection methods of NSCLC include Sanger sequencing, fluorescence *in situ* hybridization (FISH), real-time PCR (qRT-PCR), immunohistochemistry (IHC), next-generation sequencing (NGS), etc. In clinical treatment, patients can take appropriate tests to determine their carcinogenic drivers. If Sanger sequencing is chosen, DDR mutation, PTEN mutation can be detected. If FISH is chosen, NRGs fusion can be detected. If IHC is chosen, PTEN can be detected. If NGS is chosen, NRGs fusion, KRAS G12C mutation, STK11/LKB1 mutation, NFE2L2/KEAP1 mutation, RICTOR amplification, etc. can be detected. Other tests are also useful in identifying cancer-causing targets, such as whole exome sequencing for RASGRF1 fusion and RICTOR amplification, whole-transcriptome sequencing for CLIP1-LTK fusion, and RNA sequencing for LINE-1-FGGY. Moreover, re-detection of oncogenic drivers after failure of existing targeted therapies recommends the use of NGS to detect their resistance mechanisms.

With advances in genetic testing, new oncogenic drivers in non-small cell lung cancer are being discovered, and drugs developed to attenuate these new targets may be applied not only as therapeutics but also to drug-resistant cells as sensitizing treatments. Some of the characteristics of these emerging targets are summarized in Table 2 and Table 3, in which PTEN mutation, KRAS G12C, STK11/LKB1 mutation, NFE2L2/KEAP1 mutation, RICTOR amplification, YES1 amplification occur more frequently in patients with non-small cell lung cancer, but most of these emerging targets lack marketed targeted drugs. Further research is needed to apply these targeted drugs to the clinic. In the existing targeted therapy, abnormal activation of downstream signaling pathways is one of the reasons for their treatment failure. KRAS G12C and PTEN mutation make the MAPK pathway and PI3K/AKT pathway abnormal, and there are targeted drugs for KRAS G12C mutation, which can effectively solve the treatment failure caused by RAS protein abnormalities. PTEN mutation occurs frequently in tumors and often coexists with other carcinogenic drivers, and there is currently a lack of drugs to treat this target, and restoring the function of PTEN can effectively inhibit the overactivation of the PI3K/AKT pathway. Targeted drugs against PTEN mutation are urgently needed. Some of the carcinogenesis-driven targeted drugs mentioned above have shown therapeutic effects in clinical trials. Expanding the scope of oncogenic driver detection in the clinic is conducive to individualized treatment and understanding of the drug resistance mechanisms, which can be leveraged to help NSCLC patients achieve a better prognosis.

**TABLE 2:** Features of emerging targets.

Targetable driver genes	Incidence in NSCLC	Smoking status	Correlation with other drivers	Affected signaling pathway	Agent	Drug development status
NRGs fusion	1%–2%	More in never smokers	Exclusive with other oncogenic drivers	MAPK pathwayPI3K/AKT pathway	Zenocutuzumab	On the market
					Seribantumab	On clinical Trial
					GSK2849330	On clinical Trial
					Afatinib	On the market
CLIP1-LTK fusion	0.4%	More in former smokers	Exclusive with other oncogenic drivers	MAPK pathway	Lorlatinib	On the market
				PI3K/AKT pathway		
DDR mutation	3%–4% in LUSC	unknown	Co-mutated with other oncogenic drivers	NFκB pathway	Dasatinib	On the market
				MAPK pathway	DDR1-IN-1	On preclinical trial
				PI3K/AKT pathway	KST9046	On preclinical trial
					T4H11-DM4	On preclinical trial
KRAS G12C mutation	13% in LUAD	More in smokers	Exclusive with EGFR, BRAF, ALK Frequently co-mutated with STK11,TP53	MAPK pathway	Sotorasib	On the market
				PI3K/AKT/mTOR pathway	Adagrasib	Applying for listing
					JDQ443	On clinical trial
RASGRF1 fusion	<1%	never smoker	unknown	MAPK pathway	Trametinib	On the market
				PI3K/AKT pathway	SCH772984	On preclinical trial
PTEN mutation	10%–25%	More in smokers	Co-mutated with other oncogenic drivers	PI3K/AKT pathway	NP-PTEN	On preclinical trial
				AMPK pathway		
STK11/LKB1 mutation	6%–14%	unknown	Frequently co-mutated with KRAS	AMPK pathway	Everolimus	On the market
				KEAP1/NRF2 pathway	Telaglenasta	On preclinical trial
NFE2L2/KEAP1 mutation	20% in LUAD	More in smokers	unknown	MAPK pathway	TAK-228	On clinical trial
	25%–30% in LUSC			KEAP1/NRF2 pathway	ML385	On preclinical trial
RICTOR amplification	10.3% in LUAD	never smoker	co-mutated with KRAS or EFGR	PI3K/AKT/mTOR pathway	CC-223	On clinical trial
	15.8% in LUSC		Exclusive with STK11		MLN0128	On preclinical trial
YES1 amplification	15% in LUAD	unknown	unknown	mTOR pathway	Dasatinib	On the market
	25% in LUSC				CH6953755	On preclinical trial

**TABLE 3:** Summary of emerging targets.

Emerging targets	Putative use in combination or alone	Experimental evidence	Relevant clinical trials	Relevant problems
NRGs fusion	Alone	NRGs fusion causes abnormal cell proliferation	NCT04383210	Lack of drugs targeting NRG2 fusion
CLIP1-LTK fusion	Alone	CLIP1-LTK fusion causes oncogenic transformation in NSCLC patients	Unknow	Drug lack of specificity
DDR mutation	Combination	DDR2 mutation causes poorly differentiated lung cancer cells	Unknow	Lack of drugs for clinical application
KRAS G12C mutation	Combination or alone	KRAS G12C mutation causes uncontrolled cell proliferation	NCT03785249	Few types of targeted drugs
			NCT03600883	
RASGRF1 fusion	Combination or alone	RASGRF1 fusion promotes cell transformation and tumor formation	Unknow	Lack of targeted drugs
PTEN mutation	Combination	PTEN mutation promotes cell proliferation and migration	NCT02449538	Lack of targeted drugs
STK11/LKB1 mutation	Combination	STK11/LKB1 mutation promotes tumor development	NCT02366143	Lack of targeted drugs
PELP1	Combination	PELP1 dysregulation promotes lung cancer cell proliferation	Unknow	Lack of targeted drugs
NFE2L2/KEAP1 mutation	Combination or alone	NFE2L2/KEAP1 mutation promotes tumor growth	NCT02366143	Lack of drugs for clinical application
RICTOR amplification	Combination or alone	RICTOR amplification induces malignant glioma formation	NCT01545947	Lack of targeted drugs
PINK1	Combination	PINK1 overexpression promotes lung cancer cells proliferation	NCT02697201	Lack of targeted drugs
LINE-1	Combination	LINE-1 promotes tumor growth	Unknow	Lack of targeted drugs
SphK1	Combination	SphK1 facilitates the metastasis and proliferation of NSCLC cells	Unknow	Lack of drugs for clinical application
BMI1	Combination	BMI1 causes tumorigenesis	NCT02404480	Lack of drugs for clinical application
YES1 amplification	Combination	YES1 amplification promotes cell proliferation and migration	Unknow	Lack of drugs for clinical application
